# Chest and occipito-frontal circumference measurements in the detection of low birth weight among Nigerian newborns of Igbo ethnicity

**DOI:** 10.1186/s13052-014-0081-7

**Published:** 2014-10-28

**Authors:** Ikenna K Ndu, Stella N Ibeziako, Egbuna O Obidike, Gilbert N Adimora, Benedict O Edelu, Josephat M Chinawa, Isaac N Asinobi, Nwachinemere D Uleanya

**Affiliations:** Enugu state University Teaching Hospital, Park lane, Enugu, Nigeria; University of Nigeria Teaching Hospital, Enugu, Nigeria

**Keywords:** Predictive values, Chest circumference, Occipito-frontal circumference, Nigeria, Birth weight

## Abstract

**Background:**

The World Health Organisation has recommended the use of anthropometric measurements as birth weight surrogates. However, it has been found that cut-off points for these anthropometric measurements vary across nations and ethnic groups.

**Objectives:**

To determine the predictive values of chest circumference (CC), occipito-frontal circumference (OFC) and their combinations for low birth weight (LBW) detection in Igbo newborns.

**Methods:**

Live newborns of Igbo origin were recruited within 24 hours of delivery. Their CC, OFC and weight were measured. Cut off points for predicting low birth weight was determined using ROC analysis.

**Results:**

A total of 511 live newborns were recruited. For birth weight <2500 g, cut-off values were: CC 30.9 cm; OFC 33.8 cm; summation of CC and OFC 64.9 cm; ratio of CC to OFC 0.92. For weight <2000 g, the cut-off values were: CC 29.6 cm; OFC 32.8 cm; summation of CC and OFC 63.7 cm; ratio of CC to OFC 0.91. CC correlated best with birth weight (r = 0.918).

**Conclusion:**

CC is the best predictor for LBW.

## Introduction

Birth weight is a critical determinant of survival, growth and development of the newborn and also a valuable indicator of maternal health, nutrition and quality of antenatal services [1]. Newborns weighing less than 2500 grams are described as low birth weight (LBW) and have a greater risk of morbidity and mortality [2]. Thus birth weight measurement is an important screening tool for detecting the newborn at risk with special reference to low birth weight.

More than 20 million newborns worldwide are LBW and it is the single most important underlying risk factor for neonatal deaths [3,4]. It is estimated that 90% of this global burden occurs in developing countries [5] where on the average, 58% of newborn infants are not weighed at birth [3]. The reasons adduced for this are absence of trained personnel, or that weighing scales may non-functional or unavailable at places of delivery [6-9].

This challenge notwithstanding, hospital based studies in Nigeria have shown that LBW is responsible for 63% of infant mortality as well as 45.2% of perinatal deaths and carries a 37-fold increased risk of death in the first year of life [10-12]. These findings agree with a World Health Organization (WHO) estimate that almost half of newborn mortality is associated with preterm or low birth weight babies [13].

An additional advantage of early identification of LBW babies especially in resource-poor settings is to enable prompt referral which may determine survival [14]. For practical purposes some authors recommend 2000 g as the basis for hospitalizing LBW babies [7,15]. To improve detection of LBW especially in resource-poor countries, alternative measurements have been studied in different racial groups and include chest circumference (CC) [6,15,16]^,^ occipito-frontal circumference (OFC) [17,18], mid arm circumference (MAC) [6,19] and maximum thigh circumference (MTC). CC is preferred because the landmark is easily identified and has less chance of measurement errors [6,20]. The combination of OFC and CC has also been found to be a good predictor for estimation of birth weight in view of the simplicity and non-invasiveness of measuring these two body circumferences [21].

This study was designed to correlate birth weight with CC and OFC and their combinations as summation and ratio. Their suitability in detecting potential LBW newborn babies in a predominantly Igbo ethnic group domain was determined.

## Subjects and methods

This was a hospital based, cross-sectional and descriptive multi centre study carried out in two tertiary (University of Nigeria Teaching Hospital (UNTH), Enugu, Enugu State University Teaching Hospital (ESUTH), Enugu) and one secondary health facility (Mother of Christ Specialist Hospital (MCSH), Enugu). They are all equipped with infrastructures to cater to different aspects of medicine, including Obstetrics and Paediatric practice for the state and its environs. The study was carried out between 1^st^ September and 31^st^ December 2011, at the three study centres.

Inclusion criteria include live newborns delivered at the study centers, irrespective of gestational age, sex or mode of delivery and whose parents are of Igbo tribe. Babies with gross congenital abnormalities and those whose parents refused consent were excluded in the study.

Ethical approval was obtained from the Research and Ethics Committee of the three hospitals before commencement. Informed consent was obtained from the parent/guardian of each subject before recruitment. All newborn babies who met the study criteria were recruited within the first 24 hours of delivery. The data collected included CC, OFC and weight measurements.

### Body measurements (CC, OFC and weight)

To ensure reliability and avoid inter-observer bias, all measurements were taken by one researcher alone. In addition, the anthropometric measurements were recorded before recording the birth weight to minimise potential intra-observer bias. The measurements were taken within the first 24 hours of delivery because of postnatal changes in body water composition and balance [22,23]. A particular sequence of taking measurements was adhered to: OFC first, followed by CC then weight. This was to minimise exposure time and reduce risk of hypothermia. All measurements were taken with the subject lying down.

Chest circumference was measured at the level of the nipple, at the end of expiration, to the nearest 0.1 cm using a non-elastic, flexible, fibre glass measuring tape according to standard techniques described by Forfar [24].

Occipito-frontal circumference was measured as the maximum circumference of the head to the nearest 0.1 cm with a non-elastic, flexible, fibre glass measuring tape passing above the supra-orbital ridges and over the maximum occipital prominence.

All the newborns were weighed naked on a Waymaster infant spring weighing scale to the nearest 50 grams.

Gestational age assessment was corroborated by physical assessment using the New Ballard Score [25]. Where there was discordance between the gestational age by date and the New Ballard Score, the later was used. Social classification was done using the socioeconomic index scores designed by Oyedeji [26].

### Data analysis

All the data obtained was recorded and analyzed using the Statistical Package for Social Sciences (SPSS) version 19.0 and SYSTAT version 13. Continuous variables (CC, OFC) were reported as mean and standard deviation while categorical variables were reported as the number or percentage of subjects with a particular characteristic. The combination of CC and OFC as summation and ratio were also analysed. Chi square was used to test for association between Weight categories and Sex distribution of the newborns. Continuous variables were compared using student’s t test and one-way ANOVA while prediction of birth weight by anthropometric variables was done using linear regression analysis. A p-value less than 0.05 was accepted as significant. Receiver operating characteristic curve analysis was used to identify the cut-off values for the different anthropometric measurements to predict LBW. The sensitivity, specificity and predictive values were calculated at serial cut-off points while the area under the curve was determined to evaluate the overall accuracy. Results were presented as prose, tables and figures as appropriate.

## Results

### Characteristics of study population

A total of 511 newborns who met the inclusion criteria, out of 857 newborn deliveries in the three centres within the study period. One hundred and eighty three babies (35.8%) were recruited from ESUTH, while 178 (34.8%) and 150 (29.4%) were recruited from MCSH and UNTH respectively.

There were 267 males and 244 females giving a sex ratio of 1.1:1. Fourteen percent were of LBW, 82.6% were normal birth weight while 3.3% were macrosomic. There was no significant gender difference in the weight categories (χ^2^ = 2.984, p = 0.225), see Table 1. Forty nine (9.6%) of the births were preterm, 448 (87.7%) were term, while 14 (2.7%) were post term. The birth weights (BW) of the subjects ranged from 650 g to 4500 g, with a mean BW of 3110.50 ± 617.51 g. The mean BW of the males (3205.61 ± 614.60 g) was higher than that of the female babies (3006.07 ± 604.13 g), t = 3.678, p = 0.000.

**Table 1:** **Weight categories and sex distribution of the newborns**

**Weight**	**Males**	**Females**	**Total**
	**Frequency (%)**	**Frequency (%)**	**Frequency (%)**
<2500 g	31(11.6)	41(16.8)	72(14.1)
2500-3999 g	226(84.6)	196(80.3)	422(82.6)
≥4000 g	10(3.8)	7(2.9)	17(3.3)
Total	267(100)	244(100)	511(100)

χ^2^ = 2.984, p = 0.225.

### Anthropometric parameters for different weight categories

The total mean CC was 33 ± 2.8 cm while the total mean OFC was 34.7 ± 2.0. CC/OFC (ratio) and CC + OFC (summation) had total means of 0.94 ± 0.05 and 67.8 ± 4.6 respectively. Analysis of variance showed statistically significant difference among the three weight categories with respect to the measurements Table 2.

**Table 2:** **Mean values of anthropometric variables for the different weight categories**

**Birth weight group (g)**					
**Parameter**	**<2500**	**2500-3999**	**≥4000**	**F-value**	**p-value**
	**n = 72**	**n = 422**	**n = 17**		
	**Mean ± SD**	**Mean ± SD**	**Mean ± SD**		
CC (cm)	28.10 ± 2.51	33.70 ± 1.73	38.50 ± 1.41	360.0	< 0.01
OFC (cm)	31.50 ± 2.62	35.30 ± 1.30	36.65 ± 1.06	190.1	< 0.01
CC + OFC (cm)	59.60 ± 4.94	68.94 ± 2.69	75.12 ± 2.14	329.1	< 0.01
CC/OFC ratio	0.89 ± 0.04	0.96 ± 0.04	1.05 ± 0.03	134.2	< 0.01

CC = chest circumference, OFC = Occipito-frontal circumference.

### Linear regression analysis

Four linear regression models were created, one each for CC, OFC, CC + OFC (sum) and CC/OFC (ratio) as independent variables and birth weight as dependent variable. The highest coefficient of correlation (R) and coefficient of determination (R^2^) were associated with CC followed by the sum of the circumferences, OFC and ratio of circumferences in that order. All the correlations were significant at p < 0.001. Also, the lowest standard error of the estimate (SEE) was observed with CC, followed by CC + OFC, OFC and CC/OFC ratio in that order. CC had a higher coefficient of determination (R^2^) when compared with OFC. Summation of these two variables (CC and OFC) had a higher coefficient of determination (R^2^) than OFC, however the R^2^ was lower than that of CC. The ratio of the parameters gave a coefficient of determination (R^2^) less than 0.5. The multiple regression model using CC and OFC as independent co-variables produced a higher coefficient of determination (R^2^) and lower standard error of the estimate (SEE) than any of the other four simple linear regression models (Table 3).

**Table 3:** **Comparison of four simple linear regression models and a multiple regression model**

**Variable**	**Regression equation**	**R**	**R** ^**2**^	**SEE**	**t-value**	**p-value**
CC	W = 190CC – 3204	0.916	0.839	262.12	51.6	<0.01
OFC	W = 232OFC – 4999	0.821	0.674	373.60	32.4	<0.01
Summation	W = 113SUM – 4619	0.912	0.832	268.24	50.2	<0.01
Ratio	W = 8231Ratio – 4720	0.647	0.418	498.99	19.1	<0.01
CC, OFC	W = 154CC + 53OFC – 3815	0.922	0.850	253.58	24.4	<0.01

CC = chest circumference, OFC = Occipito-frontal circumference.

**Figure 1:**
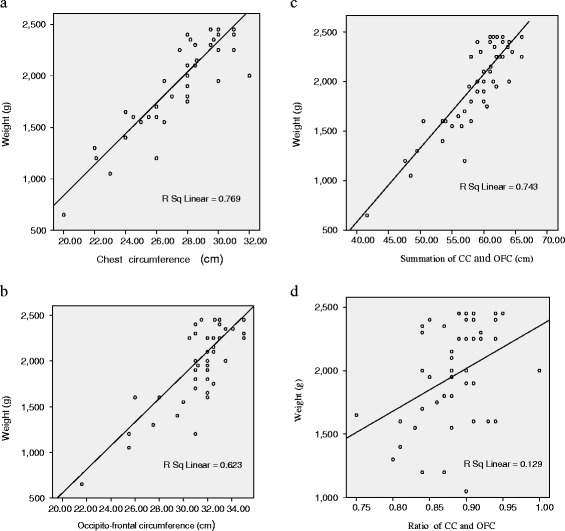
**Scatter plots/regression lines of the different anthropometric parameters for birth weight <2500g. a**: Scatter plot/regression line of birth weight (g) on chest circumference (cm) for newborns <2500 g. **b**: Scatter plot/regression line of birth weight (g) on occipitofrontal circumference for newborns <2500 g. **c**: Scatter plot/regression line of birth weight (g) on chest circumference + occipitofronal circumference (cm) for newborns <2500 g. **d**: Scatter plot/regression line of birth weight (g) on chest circumference (cc)/occipitofronal circumference (OFC) (ratio) for newborns <2500 g.

Four scatter plot graphs were created, each representing CC, OFC, CC + OFC (sum), CC/OFC (ratio) for newborns weighing less than 2500 g (Figures 1a-d). There was a linear relationship between the anthropometric measurements and birth weight as shown by the positive gradients of the scatter plot diagrams. The highest coefficient of determination (R^2^) was associated with CC followed by the sum of the circumferences, OFC and ratio of circumferences in that order.

**Figure 2:**
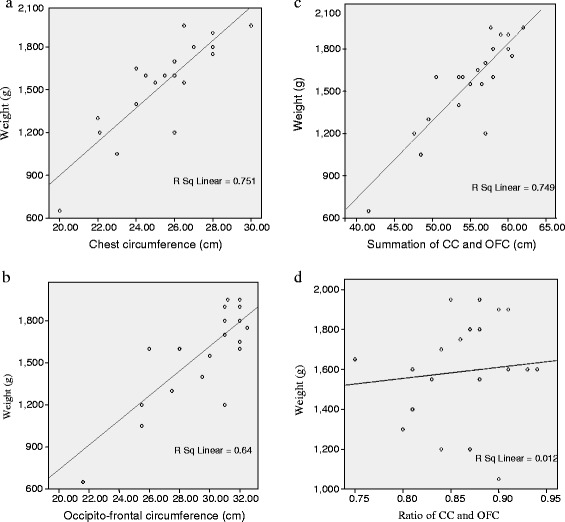
**Scatter plots/ regression lines of the different anthropometric parameters for birth weight <2000 g. a**: Scatter plot/regression line of birth weight (g) on chest circumference (cm) for newborns <2000 g. **b**: Scatter plot/regression line of birth weight (g) on occipitofronal circumference (cm) for newborns <2000 g. **c**: Scatter plot/regression line of birth weight (g) on chest circumference + occipitofronal circumference (cm) for newborns <2000 g. **d**: Scatter plot/regression line of birth weight (g) on chest circumference (CC)/occipitofronal circumference (OFC) (ratio) for newborns <2000 g.

Four scatter plot graphs were created, each representing CC, OFC, CC + OFC (sum), CC/OFC (ratio) for newborns weighing less than 2000 g (Figures 2a-d). There was a linear relationship between the anthropometric measurements and birth weight as shown by the positive gradients of the scatter plot diagrams. The highest coefficient of determination (R^2^) was associated with CC followed by the sum of the circumferences, OFC and ratio of circumferences in that order.

### ROC curve analysis for cut-off point determination

**Figure 3:**
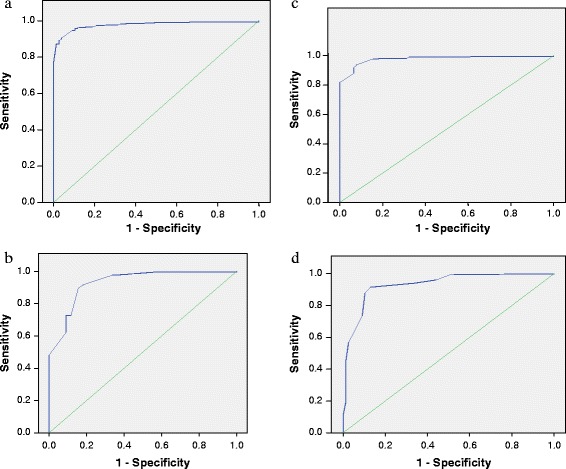
**ROC Curves of the different anthropometric parameters for birth weight <2500 g. a**: ROC curve for chest circumference as a surrogate for birth weight less than 2500 g. **b**: ROC curve for occipito-frontal circumference as a surrogate for birth weight less than 2500 g. **c**: ROC curve for chest circumference + occipitofrontal circumference as a surrogate for birth weight less than 2500 g. **d**: ROC curve for CC/OFC (ratio) as a surrogate for birth weight less than 2500 g.

Table 4 shows that both CC and summation of CC and OFC had the best discrimination for birth weight less than 2500 g. Although the AUCs for CC and summation were equal, their shapes were not identical (Figures 3a and c). In this situation, the test with the higher accuracy at the optimum cut-off points has the better discrimination. Comparing CC and CC + OFC (summation) specifically at their optimal cut-off points, CC has a higher accuracy of 94% as against 93% for summation. CC and the summation of CC and OFC gave the best discrimination for birth weight less than 2000 g. They both had equal AUCs and the same accuracy of 92%.

**Table 4:** **ROC – AUC analysis for discrimination of birth weights below 2500 g and 2000 g**

**Birth weight**	**< 2500 g (n = 72)**		**< 2000 g (n = 26)**	
**Parameter**	**AUC**	**95% CI**	**AUC**	**95% CI**
CC (cm)	0.98	0.966–0.989	0.98	0.969–0.994
OFC (cm)	0.93	0.879–0.960	0.97	0.948–0.993
CC + OFC (cm)	0.98	0.968–0.991	0.98	0.973–0.997
CC/OFC	0.93	0.899–0.963	0.93	0.897–0.967

CC = chest circumference, OFC = Occipito-frontal circumference.

The corresponding ROC curves for CC, OFC, CC + OFC and CC/OFC ratio as surrogates for birth weight less than 2500 g are shown in Figures 3a to 3d. For CC, the identified cut-off point was 30.9 cm with a sensitivity of 91.4% and {1 – specificity} of 5.3%. The optimal cut-off point for OFC was 33.8 cm with a sensitivity of 84.4% and {1 – specificity} of 10.1%. With respect to sum of circumferences, the optimal cut-off was 64.9 cm with a sensitivity of 92.2% and {1 – specificity} of 6.5%. Also, CC/OFC ratio had an optimal cut-off point of 0.92 with a sensitivity of 87.0% and {1 – specificity} of 8.5% (Table 5).

**Table 5:** **Predictive performance of selected median cut-off points of CC, OFC, summation and ratio as surrogate indices for birth weight <2500 g**

**Cut-off point (cm)**	**Sensitivity**	**Specificity**	**PPV**	**NPV**
30.9 [CC]	0.914	0.947	0.742	0.986
33.8 [OFC]	0.844	0.899	0.581	0.973
64.9 [CC + OFC]	0.922	0.935	0.702	0.986
0.92 [CC/OFC]	0.870	0.915	0.630	0.978

PPV = positive predictive, NPV = negative predictive value.

**Figure 4:**
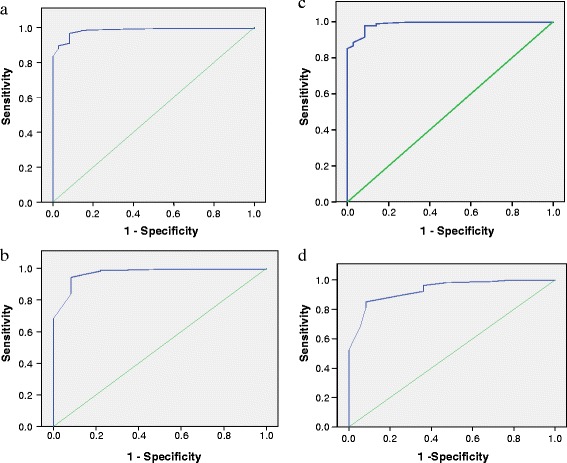
**ROC Curves of the different anthropometric parameters for birth weight <2000 g. a**: ROC curve for chest circumference as a surrogate for birth weight less than 2000 g. **b**: ROC curve for occipitofrontal circumference as a surrogate for birth weight less than 2000 g. **c**: ROC curve for chest circumference + occipitofrontal circumference as a surrogate for birth weight less than 2000 g. **d**: ROC curve for chest circumference/occipitofronal circumference (ratio) as a surrogate for birth weight less than 2000 g.

The corresponding ROC curves for CC, OFC, CC + OFC and CC/OFC ratio as surrogates for birth weight less than 2000 g are shown in Figures 4a to 4d. For CC, the identified cut-off point was 29.6 cm with a sensitivity of 91.7% and {1 –specificity} of 8.0%. The optimal cut-off point for OFC was 32.8 cm with a sensitivity of 91.7% and {1 – specificity} of 5.9%. With respect to sum of circumferences, the optimal cut-off was 63.7 cm with a sensitivity of 91.7% and {1 – specificity} of 8.2%. Also, CC/OFC ratio had an optimal cut-off point of 0.91 with a sensitivity of 75.0% and {1 – specificity} of 10.5% (Table 6).

**Table 6:** **Predictive performance of selected median cut-off points of CC, OFC, summation and ratio as surrogate indices for birth weight <2000 g**

**Cut-off point (cm)**	**Sensitivity**	**Specificity**	**PPV**	**NPV**
29.6 (CC)	0.917	0.920	0.653	0.985
32.8 (OFC)	0.917	0.919	0.647	0.985
63.7 (CC + OFC)	0.917	0.918	0.647	0.985
0.91 (CC/OFC)	0.750	0.895	0.540	0.956

PPV = positive predictive, NPV = negative predictive value.

## Discussion

Birth weight is an important screening tool for detecting the newborn at risk with special reference to LBW [1]. The LBW incidence of 14% in the current study is comparable to the estimated national average of 12% [27]. Detecting LBW is a challenge in developing countries because of unavailable or unreliable weighing scales and deliveries outside healthcare facilities [3,6,7]. This has led to the need for alternative measurements to assess newborns.

In this current study involving babies of Igbo ethnic extraction form Nigeria, birth weight correlated very strongly with the anthropometric variables of CC, CC + OFC (sum) and strongly with OFC. CC demonstrated the best correlation with birth weight. This is similar to findings by Fawcus in Zimbabwe [28] respectively. It is also in keeping with findings from studies done in Asian countries which have reported good correlation between CC and birth weight ranging from 0.790 to 0.842 [19,20,29]. The high coefficient of correlation in the current study and the other studies cited above further reinforces the recommendation of the WHO collaborative study [6] to use CC as an alternative measurement for detection of low birth weight. This strong correlation between CC and birth weight may be due to the fact that there are no significant soft tissue changes occasioned by the delivery process for CC.

OFC correlated well with birth weight, though not as strong as that of CC. Variations in the degree of moulding and oedema may be responsible for the lower correlation when compared with CC. These soft tissue changes differ from baby to baby depending on the circumstances of labour such as prolonged and obstructed labour [17]. Such variation may likely affect the correlation between OFC and birth weight.

The summation of OFC and CC had a strong correlation with birth weight, superior to OFC alone and approaching that of CC. However, the summation of OFC and CC as a surrogate to birth weight requires mathematical calculation and thus may offer no practical advantage over CC alone. No previous study on summation of OFC and CC for prediction of birth weight was found.

CC/OFC ratio had the least correlation among all the parameters analysed in the current study. Furthermore, the ratio of the parameters gave a coefficient of determination (R^2^) less than 0.5 which indicates that less than half of the variation in birth weight can be explained by CC/OFC ratio. Hence, there is no advantage in working out the ratio. It also requires calculation and may not be of much use to the semi-skilled labour attendant. No previous study was found on CC/OFC ratio for prediction of birth weight.

The multiple regression model using CC and OFC as independent co-variables explains more variations that exists in birth weight than any of the other four models and is thus the most predictive formula for birth weight calculation. However, it may have limited application in the field because of the calculations involved.

A previous study in Nigeria revealed that Igbo babies have the highest birth weights of other ethnic groups in Nigeria [30]. When compared to figures from outside Nigeria, the cut-off point for LBW in the current study was higher than values obtained by Fawcus [28] in Zimbabwe who reported 30 cm. However it is similar to the value obtained by Moshen [17] in Egypt who reported a cut off point of 31 cm and those from Asia [20,31]. The findings of the current study and other studies from both Africa [17,28] and Asia [20,31] fall between a range of 29.0 cm and 31.0 cm. This range may be considered wide enough to highlight the challenge in adopting a universal cut off for LBW.

The mean birth weight obtained in the current study is somewhat higher than that of Ezeaka et al. [18] in Lagos and Swende [32] in Makurdi who reported lower values of 2890 g and 3080 g respectively. It is however lower than the 3200 g obtained by Patwari and colleagues [30] who studied only babies from privileged backgrounds in Maiduguri, a region with comparatively lower mean birth weight.

When compared to figures reported from outside Nigeria, the mean birth weight found in the current study is substantially higher than 2364 g and 2866 g observed in India and Vietnam respectively [6]. On the other hand, it is smaller than 3300 g to 3650 g from North America and Europe [33,34]. The reason for the observed differences could range from racial and ethnic to socioeconomic factors.

Development of colour coded tapes for use by midwives and TBAs or family members will facilitate identification and referral of LBW newborns. Based on the cut-off points from this study, a colour coded tape can easily identify three weight groups. Those weighing more than 2500 g will fall within green, 2000 – 2500 g will fall the yellow area, while less than 2000 will be red.

## Conclusion

CC appears to be the best surrogate for detecting LBW infants. It is easy to measure and demonstrated the best correlation of all the parameters. This finding is in keeping with the WHO recommendation and should be encouraged in the rural areas and primary health care centres where weighing scales are likely not to be available or unreliable. Measuring tape is the only tool required and it is readily available, affordable and easily replaceable when damaged.

## Abbreviations

AUC: Area under the curve
CC: Chest circumference
ESUTH: Enugu State University Teaching Hospital
GA: Gestational age
LBW: Low birth weight
MAC: Mid arm circumference
MCSH: Mother of Christ Specialist Hospital
MTC: Maximum thigh circumference
NBSCU: Newborn special care unit
OFC: Occipitofrontal circumference
ROC: Receiver operating characteristic
SD: Standard deviation
SEE: Standard error of the estimate
SPSS: Statistical package for social sciences
TBAs: Traditional birth attendants
UNICEF: Nations Children Fund
UNTH: University of Nigeria Teaching Hospital
WHO: World Health Organisation
